# The Current State of Obstetric Nursing in Brazil

**DOI:** 10.1590/1518-8345.0000.3510

**Published:** 2021-11-19

**Authors:** Ana Paula Cavalcante de Oliveira, Carla Aparecida Arena Ventura, Mariana Lopes Galante, Mónica Padilla, Anna Cunha, Isabel Amelia Costa Mendes, Kleyde Ventura de Souza, Manoel Carlos Neri da Silva, Mayra Isabel Correia Pinheiro, Nádia Mattos Ramalho, Sonia Acioli, Vinícius Nunes Azevedo

**Affiliations:** 1 Organização Pan-Americana da Saúde/Organização Mundial da Saúde (OPAS/OMS), Unidade Técnica de Capacidades Humanas para a Saúde, Brasília, DF, Brazil.; 2 Universidade de São Paulo, Escola de Enfermagem de Ribeirão Preto, PAHO/WHO Collaborating Centre for Nursing Research Development, Ribeirão Preto, SP, Brazil.; 3 Fundo de População das Nações Unidas (UNFPA), Brasília, DF, Brazil.; 4 Grupo de Trabalho Campanha Nursing Now Brazil, Brasília, DF, Brazil.; 5 Associação Brasileira de Obstetrizes e Enfermeiros Obstetras (ABENFO) Nacional, Rio de Janeiro, RJ, Brazil.; 6 Conselho Federal de Enfermagem (COFEN), Brasília, DF, Brazil.; 7 Ministério da Saúde, Secretaria de Gestão do Trabalho e da Educação na Saúde (MS/SGTES), Brasília, DF, Brazil.; 8 Associação Brasileira de Enfermagem (ABEn Nacional), Brasília, DF, Brazil.

**Figure:**
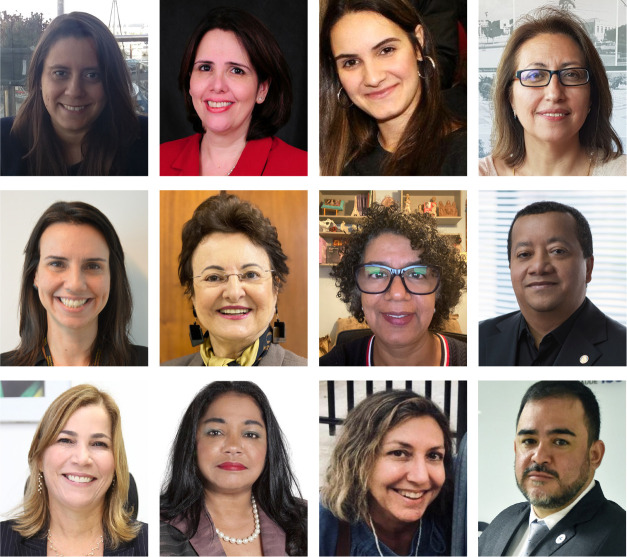


In the scope of the celebrations of the International Year of Nursing and Obstetrics (2020), two reports were organized with contributions from representatives of the World Health Organization (WHO) member countries, namely: “State of the World’s Nursing 2020” (SoWNy 2020)^(1)^ and “State of the World’s Obstetrics 2021” (SoWMy 2021)^(2)^. The SoWMy report, conducted by the United Nations Population Fund (UNFPA) in conjunction with the WHO and the International Confederation of Midwives (ICM), was launched in the context of the designation of 2021 as the International Year of Health Workers and Caregivers by the 73^rd^ World Health Assembly.

The SoWMy 2021 report documents the workforce in the provision of Sexual, Reproductive, Maternal, Neonatal and Adolescent (SRMNA) health care, including the situation of Obstetric Nursing and midwives using data collected through the National Health Workforce Accounts tool, developed by the WHO, and the questionnaire developed by ICM, UNFPA and Direct Relief, applied in more than 140 member associations. From this perspective, it seeks to contribute to mitigating the challenge faced in planning the health workforce and assessing the ability to meet the population’s health care and service needs, hampered by the deficiency of the information systems on human resources^(2)^. The report describes the important role of the professionals in the SRMNA health care area, aiming to improve health and development indicators, such as the Sustainable Development Goals (SDGs).

Continuing the collaborative work to establish the profile of Nursing in Brazil in the SoWNy 2020 report^(3)^, the Brazilian Association of Nursing (*Associação Brasileira de Enfermagem*, ABEn), the Brazilian Association of Midwives and Obstetric Nurses (*Associação Brasileira de Obstetrizes e Enfermeiros Obstetras*, ABENFO), the Federal Council of Nursing (*Conselho Federal de Enfermagem*, COFEN), the Ministries of Education and of Health, the Collaborating Center of the Pan American Health Organization (PAHO/WHO) for the Development of Research in Nursing of the Ribeirão Preto Nursing School at the University of São Paulo, the Work Group of the Nursing Now Campaign in Brazil, and the UNFPA and PAHO/WHO Brazil representation (PAHO/WHO/BRA), made a commitment to contribute to the SoWMy 2021 report and to the preparation of the infographic entitled “Photography of Obstetric Nursing in Brazil”^(4)^.

The SoWMy 2021 report grouped the workers considered as extended workforce in SRMNA health care into three subgroups: (a) expanded workforce in Obstetrics, which includes midwives with higher or mid-level education, midwives who were not classified in the previous category, and Nursing professionals trained in Obstetrics (higher and mid-level/technical); (b) Nursing workers, excluding those trained in Obstetrics; and (c) physicians who work in SRMNA health care, including general practitioners (e.g., family physician), obstetricians/gynecologists and pediatricians. Information about other professionals was also included, such as community health agents who play an important role in SRMNA health care.

The SoWMy report analyzed the workforce in 194 countries and found that, although midwives can provide 90% of the essential SRMNA health care, they represent less than 10% of the workforce. It also highlights the scarcity of these professionals, since 1.1 million workers are needed (using equivalent time of dedication in the SRMNA health care area), of which nearly one million are midwives, mainly in low-income countries^(2)^. The presence of midwives in the provision of care would prevent 67% of the maternal deaths, 64% of the newborn deaths and 65% of the stillbirth cases, saving an estimated 4.3 million lives *per* year^(2)^.

Obstetric nurses are not only present in deliveries, they provide pre- and post-natal care, as well as a variety of sexual and reproductive health services, such as family planning, detection and treatment of sexually transmitted infections, as well as sexual and reproductive health services for adolescents. The group of professionals classified as the extended workforce in Obstetrics totals 1.9 million professionals, reaching 160,000 in the Americas regions; globally, it presents a mean density of 4.4 *per* 10,000 inhabitants, the highest value being found in Southeast Asia (10.4 *per* 10,000 inhabitants)^(2)^. In the Americas region, density is 1.9 *per* 10,000 inhabitants (excluding the United States of America, this density is now 2.9), noting that Cuba presents the highest density in the world, with 46.89. The most worrying shortage is in the African region (56% of the overall shortage), followed by the East Mediterranean and Americas regions^(2)^. In low- and middle-income countries, these professionals collaborate to achieve significant reductions in maternal and neonatal mortality, as well as in stillbirths, although they need to be properly trained to carry out these activities^(5)^.

According to the data collected in 80 countries, training in Obstetrics is characterized by programs that are distributed as follows: 33 (41%) countries only offer direct entry programs (degree in Obstetrics), 17 (21%) offer only graduate courses in Nursing (Obstetric Nurse), five (6%) offer combined Nursing and Obstetrics degree programs, and 25 (31%) offer direct entry and other type of training programs^(2)^.

In Brazil, as all Nursing professionals can provide care in the SRMNA health care area, as long as they are registered with the Nursing Council for practicing the profession, and based on the context indicated above about the training of midwives, in this editorial, the “extended workforce in Obstetrics” is considered as a “workforce in Obstetric Nursing”, comprised by nurses, in general, and by those with training in Obstetrics, as well as by nursing assistants and technicians (mid-level professionals). In this context, it is noteworthy that, in addition to educational programs - specialization in Obstetric Nursing or residency in Obstetric Nursing - ABENFO can also certify and qualify professionals as specialists, based on proof of title and proof of assistance activities, among other criteria. Data indicate a total of 1,561,940 professionals (number of professionals with active registration until May 2020), with 405,961 nurses with a density of 19.32 *per* 10,000 inhabitants^*^ (considering generalist nurses and all specializations); 2,049 obstetric nurses^**^, with a density of 0.10; and 1,155,979 nursing technicians and assistants, with a density of 55.00^(2)^. It is estimated that the Brazilian population will grow 12%, totaling 222.7 million people by 2030. Therefore, the Obstetrics services will have to serve 4.5 million pregnancies a year until 2030 in order to ensure universal access to SRMNA health care^(6)^.

With 99.10% of the deliveries assisted by qualified health professionals and 91% coverage of prenatal care (minimum of four appointments), Brazil presents improved indicators on the outcome of the care provided in the area and impacts generated on the health of mothers and newborns. As examples, the neonatal mortality rate (deaths within 28 days *per* 1,000 live births) was reduced from 17.98 in 2000 to 8 in 2020. However, challenges still persist such as maternal mortality and a high cesarean rate of 56%, especially considering the global mean of 21%^(2)^ (the WHO›s recommendation is that up to 15% of the deliveries be by cesarean section).

The COVID-19 pandemic brought to light challenges to be overcome by the health systems, such as interruptions in the SRMNA health care services^(7)^, workforce availability and more effective performance, specifically for the Obstetrics workforce, which, as identified in the SoWMy report, is scarce worldwide. From this perspective, it is necessary to reinforce actions to strengthen it, considering the consequences of the impact of the pandemic on this workforce, mostly made up of women (88% of the Nursing workforce in Brazil). These professionals are suffering intensely from the consequences, especially in terms of job security, increased responsibilities related to care and remote education, as well as the increased incidence of gender-based violence^(2)^. The demands for assistance and quality of the SRMNA health care services, as a priority area in global public health, generate important challenges to the complementary development of the different professions associated with this process in the fields of training, regulation and empowerment of the professional practice, all this in response to the particularities of each country.

For these professionals to reach their potential, investments in education are needed; as well as health workforce planning, management and regulation, with information systems that incorporate data on these professionals and their working conditions; scientific research, knowledge production and innovation in the practice; and leadership and governance^(2)^, highlighting the participation of these professionals in the elaboration of health policies and improving the development of their leadership potential in facing the challenge of expanding access to safe, quality and resolute care for women. Such investment will result in improved availability, accessibility, acceptability and quality of the workforce, reflecting positive impacts on people’s health and gender equality, which may generate an increase in the labor offer, as well as a positive macroeconomic impact for the country^(2)^.

Particularly in Brazil, the data analysis highlighted elements to drive the discussion in the area and assist in the design and implementation of public policies. We hope that this information can also be used for the projection and sustainability of the agendas, allowing for the expansion of access and coverage of the services, as well as strengthening of the Unified Health System towards Universal Health.

## Footnotes

* Brazilian population in 2019: 210,147,125. Source: IBGE- Population Estimates (Estimate for the TCU, available through DATASUS/Tabnet).

** This number refers to Nursing professionals registered with the Nursing Council with a specialization in Obstetrics (using the root of the word to identify the professionals in the database).
